# Supplementary material for: MEME-LaB: motif analysis in clusters
Source: Bioinformatics. 2013 May 14;29(13):1696–7. doi: 10.1093/bioinformatics/btt248 (PMC3694638; doi:10.1093/bioinformatics/btt248)
Supplement: Supplementary Data [file supp_btt248_Supplementary_Data.zip › Supplementary_Data/Results_Files/which_gene_ids/cluster15_motif1.html]

Cluster 15 Motif 1


Cluster 15 Motif 1 is found in the following promoters

| Gene ID | Strand | Position\* |
| --- | --- | --- |

|  |  |  |
| --- | --- | --- |
| AT1G01140 | + | 46/500 |
| AT1G01920 | - | 399/500 |
| AT1G02330 | + | 151/500 |
| AT1G04770 | - | 189/500 |
| AT1G05170 | - | 146/500 |
| AT1G05200 | - | 111/500 |
| AT1G05350 | - | 178/500 |
| AT1G06380 | + | 399/500 |
| AT1G06670 | - | 461/500 |
| AT1G08820 | + | 243/500 |
| AT1G11545 | + | 474/500 |
| AT1G11780 | + | 445/500 |
| AT1G12120 | - | 265/500 |
| AT1G12650 | - | 479/500 |
| AT1G13260 | - | 241/500 |
| AT1G13980 | + | 292/500 |
| AT1G16830 | + | 45/500 |
| AT1G16870 | + | 22/500 |
| AT1G17610 | + | 439/500 |
| AT1G17680 | + | 109/500 |
| AT1G18710 | - | 296/500 |
| AT1G18800 | - | 180/500 |
| AT1G18850 | + | 329/500 |
| AT1G18950 | + | 236/500 |
| AT1G19100 | + | 416/500 |
| AT1G21750 | - | 274/500 |
| AT1G24320 | + | 146/500 |
| AT1G24460 | - | 475/500 |
| AT1G26130 | - | 216/500 |
| AT1G26260 | - | 431/500 |
| AT1G26270 | + | 361/500 |
| AT1G26640 | + | 489/500 |
| AT1G28530 | + | 30/500 |
| AT1G29320 | + | 311/500 |
| AT1G29390 | + | 311/500 |
| AT1G32440 | + | 32/500 |
| AT1G32700 | + | 363/500 |
| AT1G34380 | + | 115/500 |
| AT1G35730 | - | 446/500 |
| AT1G43860 | - | 453/500 |
| AT1G45688 | - | 231/500 |
| AT1G47710 | - | 267/500 |
| AT1G48760 | + | 121/500 |
| AT1G49600 | + | 260/500 |
| AT1G49820 | + | 338/500 |
| AT1G51540 | + | 50/500 |
| AT1G52160 | - | 488/500 |
| AT1G52410 | + | 188/500 |
| AT1G53830 | + | 417/500 |
| AT1G54050 | - | 146/500 |
| AT1G54770 | - | 342/500 |
| AT1G60850 | - | 201/500 |
| AT1G61380 | - | 193/500 |
| AT1G62390 | - | 75/500 |
| AT1G63660 | - | 271/500 |
| AT1G63780 | - | 405/500 |
| AT1G64980 | + | 370/500 |
| AT1G65660 | + | 241/500 |
| AT1G65900 | + | 436/500 |
| AT1G67170 | - | 353/500 |
| AT1G67470 | + | 233/500 |
| AT1G68185 | - | 64/500 |
| AT1G68990 | + | 206/500 |
| AT1G69010 | - | 74/500 |
| AT1G70060 | - | 193/500 |
| AT1G71210 | + | 344/500 |
| AT1G71850 | - | 224/500 |
| AT1G72050 | + | 451/500 |
| AT1G73770 | - | 78/500 |
| AT1G73805 | - | 468/500 |
| AT1G74430 | + | 439/500 |
| AT1G74710 | + | 359/500 |
| AT1G75370 | + | 292/500 |
| AT1G75510 | - | 462/500 |
| AT1G76270 | - | 89/500 |
| AT1G76580 | + | 222/500 |
| AT1G76710 | + | 468/500 |
| AT1G76820 | - | 371/500 |
| AT1G78070 | - | 396/500 |
| AT1G79690 | + | 415/500 |
| AT1G80270 | + | 340/500 |
| AT1G80770 | + | 474/500 |
| AT1G80930 | - | 278/500 |
| AT2G02570 | - | 486/500 |
| AT2G03140 | - | 381/500 |
| AT2G06925 | + | 355/500 |
| AT2G07739 | + | 477/500 |
| AT2G13370 | - | 487/500 |
| AT2G14520 | - | 246/500 |
| AT2G16365 | - | 475/500 |
| AT2G16380 | + | 433/500 |
| AT2G16860 | - | 196/500 |
| AT2G17560 | - | 221/500 |
| AT2G18220 | + | 456/500 |
| AT2G19690 | + | 54/500 |
| AT2G19780 | - | 432/500 |
| AT2G20020 | + | 305/500 |
| AT2G20340 | + | 443/500 |
| AT2G20490 | - | 285/500 |
| AT2G21370 | + | 438/500 |
| AT2G21380 | - | 91/500 |
| AT2G21730 | + | 484/500 |
| AT2G21790 | + | 98/500 |
| AT2G22300 | + | 473/500 |
| AT2G22360 | + | 480/500 |
| AT2G22400 | + | 309/500 |
| AT2G23120 | + | 47/500 |
| AT2G23890 | + | 36/500 |
| AT2G24330 | + | 222/500 |
| AT2G25355 | + | 348/500 |
| AT2G26300 | - | 308/500 |
| AT2G28480 | - | 181/500 |
| AT2G28890 | + | 124/500 |
| AT2G31240 | + | 441/500 |
| AT2G31880 | + | 405/500 |
| AT2G32070 | + | 327/500 |
| AT2G34260 | + | 444/500 |
| AT2G34570 | + | 224/500 |
| AT2G34940 | - | 247/500 |
| AT2G35190 | - | 203/500 |
| AT2G35540 | - | 434/500 |
| AT2G37710 | - | 471/500 |
| AT2G38300 | - | 110/500 |
| AT2G38550 | - | 85/500 |
| AT2G38590 | + | 466/500 |
| AT2G39900 | + | 474/500 |
| AT2G40750 | + | 20/500 |
| AT2G41670 | - | 362/500 |
| AT2G42450 | - | 270/500 |
| AT2G43210 | - | 109/500 |
| AT2G44200 | - | 55/500 |
| AT2G44490 | + | 342/500 |
| AT2G45560 | - | 101/500 |
| AT2G45660 | - | 484/500 |
| AT2G45860 | + | 214/500 |
| AT2G47240 | + | 359/500 |
| AT2G47470 | - | 456/500 |
| AT3G01160 | - | 402/500 |
| AT3G01790 | - | 276/500 |
| AT3G02630 | + | 453/500 |
| AT3G03490 | - | 326/500 |
| AT3G03960 | - | 165/500 |
| AT3G04110 | + | 425/500 |
| AT3G04480 | + | 329/500 |
| AT3G04920 | - | 80/500 |
| AT3G05270 | + | 146/500 |
| AT3G05830 | - | 202/500 |
| AT3G06550 | + | 390/500 |
| AT3G07990 | + | 162/500 |
| AT3G08510 | + | 114/500 |
| AT3G08970 | - | 387/500 |
| AT3G09720 | + | 212/500 |
| AT3G11090 | - | 380/500 |
| AT3G11450 | + | 166/500 |
| AT3G11964 | - | 235/500 |
| AT3G13230 | - | 140/500 |
| AT3G13580 | - | 186/500 |
| AT3G13940 | + | 395/500 |
| AT3G15080 | - | 325/500 |
| AT3G15730 | - | 57/500 |
| AT3G15940 | + | 420/500 |
| AT3G16350 | - | 454/500 |
| AT3G16810 | - | 488/500 |
| AT3G17120 | + | 155/500 |
| AT3G19630 | - | 154/500 |
| AT3G20470 | - | 462/500 |
| AT3G21080 | + | 275/500 |
| AT3G22300 | + | 120/500 |
| AT3G23620 | - | 188/500 |
| AT3G25230 | - | 478/500 |
| AT3G25940 | + | 199/500 |
| AT3G27280 | - | 386/500 |
| AT3G29270 | - | 210/500 |
| AT3G43590 | - | 477/500 |
| AT3G43960 | - | 133/500 |
| AT3G44735 | + | 287/500 |
| AT3G46290 | - | 382/500 |
| AT3G46630 | + | 380/500 |
| AT3G47910 | - | 487/500 |
| AT3G48280 | + | 395/500 |
| AT3G48610 | - | 402/500 |
| AT3G48710 | - | 487/500 |
| AT3G49340 | + | 2/500 |
| AT3G49725 | + | 152/500 |
| AT3G49990 | + | 266/500 |
| AT3G50480 | - | 298/500 |
| AT3G52220 | - | 350/500 |
| AT3G52870 | + | 400/500 |
| AT3G54980 | - | 453/500 |
| AT3G55005 | - | 468/500 |
| AT3G55510 | - | 386/500 |
| AT3G55605 | - | 6/500 |
| AT3G56070 | - | 68/500 |
| AT3G56400 | - | 365/500 |
| AT3G56680 | + | 479/500 |
| AT3G56720 | + | 130/500 |
| AT3G57660 | + | 324/500 |
| AT3G57940 | - | 80/500 |
| AT3G59890 | + | 305/500 |
| AT3G59970 | - | 125/500 |
| AT3G60210 | - | 269/500 |
| AT3G60810 | + | 369/500 |
| AT3G61280 | - | 209/500 |
| AT3G62620 | + | 264/500 |
| AT3G62630 | + | 385/500 |
| AT3G62690 | + | 264/500 |
| AT4G00620 | + | 424/500 |
| AT4G00970 | + | 119/500 |
| AT4G01550 | + | 333/500 |
| AT4G01915 | - | 20/500 |
| AT4G02230 | + | 189/500 |
| AT4G02500 | - | 213/500 |
| AT4G03250 | - | 389/500 |
| AT4G10620 | - | 452/500 |
| AT4G11790 | - | 455/500 |
| AT4G12010 | - | 345/500 |
| AT4G12460 | - | 231/500 |
| AT4G12600 | - | 292/500 |
| AT4G12750 | + | 21/500 |
| AT4G12900 | + | 68/500 |
| AT4G13040 | - | 326/500 |
| AT4G13070 | + | 292/500 |
| AT4G13345 | + | 450/500 |
| AT4G15640 | + | 308/500 |
| AT4G16155 | - | 280/500 |
| AT4G16860 | - | 134/500 |
| AT4G16940 | - | 461/500 |
| AT4G17100 | - | 414/500 |
| AT4G18390 | - | 124/500 |
| AT4G20400 | - | 489/500 |
| AT4G20890 | + | 208/500 |
| AT4G21570 | - | 216/500 |
| AT4G23180 | + | 225/500 |
| AT4G25315 | + | 420/500 |
| AT4G25630 | - | 195/500 |
| AT4G26055 | + | 465/500 |
| AT4G27340 | + | 19/500 |
| AT4G29510 | + | 489/500 |
| AT4G30080 | - | 226/500 |
| AT4G31500 | - | 2/500 |
| AT4G32190 | + | 288/500 |
| AT4G33060 | + | 371/500 |
| AT4G34620 | + | 469/500 |
| AT4G36150 | - | 142/500 |
| AT4G36680 | + | 489/500 |
| AT4G36970 | - | 302/500 |
| AT4G37440 | - | 134/500 |
| AT4G37910 | + | 103/500 |
| AT4G38270 | - | 404/500 |
| AT4G38620 | + | 197/500 |
| AT4G38890 | + | 301/500 |
| AT4G39350 | - | 476/500 |
| AT5G01750 | - | 151/500 |
| AT5G04360 | + | 488/500 |
| AT5G04600 | + | 221/500 |
| AT5G04670 | - | 190/500 |
| AT5G06550 | + | 174/500 |
| AT5G06610 | + | 119/500 |
| AT5G06950 | + | 186/500 |
| AT5G07870 | + | 489/500 |
| AT5G08185 | - | 186/500 |
| AT5G08630 | + | 378/500 |
| AT5G09270 | - | 226/500 |
| AT5G10290 | + | 321/500 |
| AT5G10490 | - | 156/500 |
| AT5G10550 | + | 316/500 |
| AT5G12900 | + | 393/500 |
| AT5G13560 | + | 291/500 |
| AT5G13680 | + | 357/500 |
| AT5G14270 | - | 200/500 |
| AT5G14600 | + | 325/500 |
| AT5G14720 | - | 24/500 |
| AT5G14970 | + | 470/500 |
| AT5G15700 | - | 225/500 |
| AT5G16680 | + | 183/500 |
| AT5G17010 | - | 233/500 |
| AT5G17270 | + | 200/500 |
| AT5G17620 | - | 429/500 |
| AT5G19300 | + | 192/500 |
| AT5G20170 | + | 287/500 |
| AT5G21140 | - | 141/500 |
| AT5G21160 | + | 483/500 |
| AT5G21900 | - | 457/500 |
| AT5G22100 | + | 395/500 |
| AT5G22330 | + | 253/500 |
| AT5G22370 | - | 390/500 |
| AT5G23535 | + | 362/500 |
| AT5G23580 | - | 486/500 |
| AT5G23860 | + | 6/500 |
| AT5G24660 | + | 401/500 |
| AT5G24840 | - | 485/500 |
| AT5G25440 | + | 112/500 |
| AT5G27395 | - | 7/500 |
| AT5G27620 | - | 359/500 |
| AT5G35840 | - | 86/500 |
| AT5G39790 | + | 114/500 |
| AT5G40200 | + | 120/500 |
| AT5G40570 | + | 274/500 |
| AT5G41020 | - | 257/500 |
| AT5G41460 | + | 107/500 |
| AT5G42060 | - | 274/500 |
| AT5G43950 | + | 14/500 |
| AT5G44400 | - | 451/500 |
| AT5G44582 | + | 487/500 |
| AT5G45250 | + | 405/500 |
| AT5G46020 | - | 359/500 |
| AT5G46550 | + | 139/500 |
| AT5G48380 | + | 456/500 |
| AT5G48680 | + | 366/500 |
| AT5G51660 | + | 309/500 |
| AT5G52550 | - | 488/500 |
| AT5G53280 | + | 189/500 |
| AT5G54710 | + | 185/500 |
| AT5G55960 | - | 429/500 |
| AT5G56010 | + | 218/500 |
| AT5G56250 | + | 393/500 |
| AT5G59000 | - | 454/500 |
| AT5G60890 | - | 432/500 |
| AT5G60900 | + | 135/500 |
| AT5G61020 | + | 314/500 |
| AT5G63320 | - | 475/500 |
| AT5G64420 | + | 151/500 |
| AT5G64470 | + | 71/500 |
| AT5G65205 | - | 212/500 |
| AT5G65670 | - | 165/500 |
| AT5G65910 | - | 171/500 |
| AT5G67320 | + | 335/500 |
| AT5G67470 | - | 454/500 |
| AT5G67630 | + | 260/500 |

**\*** Overlapping sequences are merged to avoid redundancy. As a result some sequences in the final set may be longer than the specified maximum.

Distribution

+

-

Position values represent number of bases from the 5 prime end of the sequence provided to MEME and the length of that sequence. It is these figures expressed as proportions that are used for statistical analysis.

Probability value of comparison of these values to a linear distribution, p = 0.00019

The motif is found 171 times on the + stand and 158 times on the - strand.

Probability value that it is evenly distributed between the two strands, p = 0.25

Move the mouse pointer over the gene ids to highlight the corresponding marker
